# Implementation of GeneXpert HIV-1 VL assay for intrapartum decision-making in Saskatchewan

**DOI:** 10.1128/jcm.01078-26

**Published:** 2026-09-30

**Authors:** Josephine Rybchuk, Claire Carson, Amanda Lang, Rupesh Chawla, Stephen Sanche, Camille Hamula, Ninad Mehta

**Affiliations:** 1 Department of Pathology and Laboratory Medicine, College of Medicine, University of Saskatchewan 7235, Saskatoon, Saskatchewan, Canada; 2 Division of Clinical Microbiology, Saskatchewan Health Authority 7234, Saskatoon, Saskatchewan, Canada; 3 Roy Romanow Provincial Laboratory, Saskatchewan Health Authority 6842, Regina, Saskatchewan, Canada; 4 Division of Paediatric Infectious Diseases, Jim Pattison Children's Hospital, Saskatchewan Health Authority 7234, Saskatoon, Saskatchewan, Canada; 5 Division of Infectious Diseases Department of Medicine, University of Saskatchewan 7235, Saskatoon, Saskatchewan, Canada; Mayo Clinic Minnesota, Rochester, Minnesota, USA

**Keywords:** maternal-fetal health, molecular, HIV

## LETTER

Human immunodeficiency virus (HIV) maternal-to-fetal vertical transmission risk remains high in populations with limited prenatal care (1–3). Clinical screening can reduce transmission through early detection of high viral loads (VL) (in copies/mL), where VL testing may influence treatment decisions post-antibody (Ab) screen positive. Per recent (2025) National Institutes of Health guidelines, women with VL > 1,000 should receive specific care (Table 1), including intrapartum IV zidovudine (AZT), and enhanced preventative measures against fetal infection, such as scheduled cesarean sections (4). For lower VL, results may guide appropriate intra- and post-partum antiretroviral treatments (ART) by avoiding excessive use (single-drug vs triple-drug therapies).

**TABLE 1:** Corresponding neonatal clinical decisions based on HIV VL results within 4 weeks of delivery^*a*^

Reported test result (viral load)	Treatment decision for newborn	Considerations for the mother
<50 copies/mL	Single drug therapy (AZT)	Continue current antiretrovirals; intrapartum AZT prophylaxis not required if patient ART compliant since VL tested
<1,000 copies/mL	Triple drug therapy (AZT, lamivudine, and nevirapine)	Consider Intrapartum AZT prophylaxis
>1,000 copies/mL	Triple drug therapy (AZT, lamivudine, and nevirapine)	Intrapartum AZT prophylaxis and scheduled C-section at 38 weeks recommended to minimize perinatal transmission

^*a*^ Clinical categories established according to NIH guidelines (Department of Health and Human Services [4]).

Saskatchewan, a large, sparsely populated province, has the highest Canadian HIV incidence over the last 2 decades (5). Women with high risk of infection often do not seek prepartum care, while others with known HIV+ status face obstacles accessing regular testing. Therefore, there is growing demand for near-patient (decentralized) HIV VL testing in hospitals providing labor and delivery services to inform care. On-site testing can improve access for hard-to-reach populations, facilitating appropriate interventions and tailored care plans (6). Accurate, rapid testing should translationally improve overall ward patient health.

**Fig 1:**
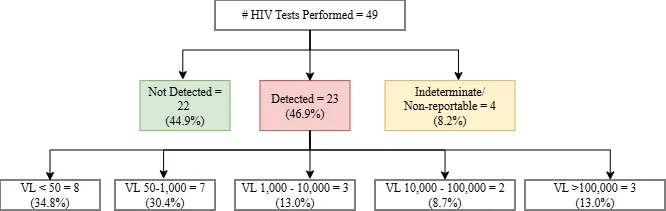
Of 49 female HIV viral-load tests in EDTA-plasma specimens, 23 had HIV detected, 22 had HIV not detected, and four generated unresolved/non-reportable GeneXpert results. All four unresolved GeneXpert results were referred for Roche cobas HIV-1 viral-load testing at the provincial reference laboratory and were target not detected. VL results are in copies/mL.

Here, we present Health Canada-approved GeneXpert HIV-1 VL XC secondary-use testing to aid clinical decision-making. Mothers admitted to Jim Pattison Children’s Hospital and Regina General Hospital with no prenatal care, recent HIV risk, and/or known HIV+ status without recent VL results were tested at physician discretion. From 2022 to 2026, maternal EDTA-plasma was tested onsite by microbiology technologists within 2 h of laboratory receipt, with a concurrent specimen sent to Roy Romanow Provincial Laboratory for Roche cobas HIV-1 NAAT (VL) testing. Detected GeneXpert results were reported in VL categories: <50; 50–1,000; 1,000–10,000; 10,000–100,000; and >100,000 copies/mL. Utilization of the GeneXpert HIV-1 test proved highly clinically viable. HIV detection occurred in 23 patients and guided appropriate treatment (see Table S1 in the supplemental material). Four non-reportable results were identified: three screening false positives, while one was successfully rerun (Fig. 1). Non-reportable results, often contamination-related or VLs below detectable limits, required follow-up Roche testing. All re-tested indeterminates were further not detected. Alignment with Roche data corroborates studies indicating success for near-patient use of HIV-1 RNA testing (7, 8). In summation, the GeneXpert had 91.84% agreement to Roche results (see Table S2 in the supplemental material). Clinical management was based on screening results and HIV status, risk assessment, and follow-up testing when required.

This study appears to be the first to describe the use of a quantitative HIV-1 VL test near-patient to inform prophylaxis. This potentially eliminates usage of unnecessary empiric treatment in 40.0% of patients with undetected VL results, and in up to 55.6% if results <50 copies/mL are included. Better testing improves mother and child outcomes by protecting fetal health and reducing side effects from inappropriate treatment decisions(9). Concordance with provincial testing enhances confidence in GeneXpert testing, and this model will be extended to in-province rural hospitals.

## SUPPLEMENTAL MATERIAL

Supplemental material jcm.01078-26-s0001.docx Tables S1 and S2.

ASM does not own the copyrights to Supplemental Material that may be linked to, or accessed through, an article. The authors have granted ASM a non-exclusive, world-wide license to publish the Supplemental Material files. Please contact the corresponding author directly for reuse.

## References

[1] Drake AL, Wagner A, Richardson B, John-Stewart G. 2014. Incident HIV during pregnancy and postpartum and risk of mother-to-child HIV transmission: a systematic review and meta-analysis. PLoS Med 11:e1001608. doi:10.1371/journal.pmed.1001608 PMC3934828 24586123

[2] European collaborative study. 1992. Risk factors for mother-to-child transmission of HIV-1. The Lancet 339:1007–1012. doi:10.1016/0140-6736(92)90534-A 1349050

[3] Levy JA. 1993. Pathogenesis of human immunodeficiency virus infection. Microbiol Rev 57:183–289. doi:10.1128/mr.57.1.183-289.1993 PMC372905 8464405

[4] Services H. 2025. Recommendations for the use of antitretroviral drugs during pregnancy and interventions to reduce perinatal HIV transmission in the United States. *In* Panel on treatment of HIV during pregnancy and prevention of perinatal transmission

[5] Spence C, Morin E, Mary Z. 2024. Saskatoon HIV program evaluation. Final Report. Saskatoon HIV Program Review

[6] Sutton M, Anthony M-N, Vila C, McLellan-Lemal E, Weidle PJ. 2010. HIV testing and HIV/AIDS treatment services in rural counties in 10 southern states: service provider perspectives. J Rural Health 26:240–247. doi:10.1111/j.1748-0361.2010.00284.x 20633092

[7] Chaplin B, Agbaji O, Reyes Nieva H, Olatunde B, Chang C, Mitruka K, Sule H, Dajel T, Zee A, Ahmed ML, Ahmed I, Okonkwo P, Rawizza H, Kanki P. 2023. Timeliness of point-of-care viral load results improves human immunodeficiency virus monitoring in Nigeria. Clin Infect Dis 76:e671–e680. doi:10.1093/cid/ciac609 PMC11150521 35872644

[8] Luo R, Fong Y, Boeras D, Jani I, Vojnov L. 2022. The clinical effect of point-of-care HIV diagnosis in infants: a systematic review and meta-analysis. The Lancet 400:887–895. doi:10.1016/S0140-6736(22)01492-1 36116479

[9] Schlösser R, Linde R, Dunsch D, Reitter A, Haberl A, Bauer K. 2007. Unerwünschte wirkungen der antiretroviralen prophylaxe der HIV-transmission bei frühgeborenen unter 35 schwangerschaftswochen. Z Geburtshilfe Neonatol 211:230–235. doi:10.1055/s-2007-981405 18176903

