# Supplementary material for: Implementation of GeneXpert HIV-1 VL assay for intrapartum decision-making in Saskatchewan
Source: J Clin Microbiol. 2026 Sep 30;64(11):e01078-26. doi: 10.1128/jcm.01078-26 (PMC13753810; doi:10.1128/jcm.01078-26)
Supplement: Supplementary file 1 [file jcm.01078-26-s0001.docx]

Supplemental Material

*Table S1.* HIV viral load comparison between the GeneXpert® HIV-1 VL XC qPCR and Roche Cobas HIV-1 NAAT for positive patients. For the GeneXpert® assay, the instrument reports quantitative viral loads within its measurement range, results below the quantifiable range, HIV not detected, or a non-reportable result such as invalid/error/no result. For the purpose of clinical communication in this study, positive results were grouped into categories that aligned with intrapartum management thresholds.

| Sr. No. | Result Reported to Patient Chart (GeneXpert®) | GeneXpert® HIV-1 VL XC (Copies/ml) | Roche cobas® HIV-1 (Copies/ml) | Previous Screen Result | Treatment Decision |
| --- | --- | --- | --- | --- | --- |
| 1 | HIV Detected - Viral Load 50 - 1,000 Copies/mL | 661 | 456 | Reactive (DUO Screen) | AZT |
| 2 | Indeterminate | N/A | Target Not Detected | Reactive (DUO Screen) | N/A |
| 3 | HIV Not Detected | Not Detected | Target Not Detected | Previous Known HIV Positive | N/A |
| 4 | HIV Not Detected | Not Detected | Target Not Detected | Negative | N/A |
| 5 | HIV Not Detected | Not Detected | Target Not Detected | Previous Known HIV Positive | N/A |
| 6 | HIV Detected - Viral Load >100,000 Copies/mL | 880000 | 669000 | Reactive (DUO Screen) | Maternal triple drug ART, C-section, infant AZT prophylaxis |
| 7 | HIV Detected - Viral Load 50 - 1,000 Copies/mL | 218 | 442 | Reactive (DUO Screen) | AZT |
| 8 | HIV Detected - Viral Load 50 - 1,000 Copies/mL | 259 | 241 | Reactive (DUO Screen) | AZT |
| 9 | HIV Detected - Viral Load 50 - 1,000 Copies/mL | <40 | 200 | Positive (Ag/Ab Combo) | AZT |
| 10 | HIV Not Detected | Not Detected | Target Not Detected | Negative | N/A |
| 11 | HIV Not Detected | Not Detected | Target Not Detected | Reactive (DUO Screen) | N/A |
| 12 | HIV Not Detected | Not Detected | Target Not Detected | Previous Known HIV Positive | N/A |
| 13 | HIV Not Detected | Not Detected | Target Not Detected | Negative | N/A |
| 14 | HIV Not Detected | Not Detected | Target Not Detected | Negative | N/A |
| 15 | HIV Not Detected | Not Detected | Target Not Detected | Negative | N/A |
| 16 | HIV Not Detected | Not Detected | Target Not Detected | Negative | N/A |
| 17 | HIV Not Detected | Not Detected | Target Not Detected | Negative | N/A |
| 18 | HIV Not Detected | Not Detected | Target Not Detected | Negative | N/A |
| 19 | HIV Detected - Viral Load >100,000 Copies/mL | 963000 | 1460000 | Reactive (DUO Screen) | Maternal triple drug ART, C-section, infant AZT prophylaxis |
| 20 | HIV Detected - Viral Load 50 - 1,000 Copies/mL | 442 | 637 | Reactive (DUO Screen) | AZT |
| 21 | HIV Not Detected | Not Detected | Target Not Detected | Reactive (DUO Screen) | N/A |
| 22 | HIV Not Detected | Not Detected | Target Not Detected | Negative | N/A |
| 23 | HIV Not Detected | Not Detected | Target Not Detected | Negative | N/A |
| 24 | HIV Detected - Viral Load <50 Copies/mL | <40 | 43 | Previous Known HIV Positive | AZT |
| 25 | Indeterminate | N/A | Target Not Detected | Negative | N/A |
| 26 | Indeterminate | N/A | Target Not Detected | Negative | N/A |
| 27 | HIV Detected - Viral Load <50 Copies/mL | <40 | 44.6 | Previous Known HIV Positive | AZT |
| 28 | HIV Not Detected | Not Detected | Target Not Detected | Reactive (DUO Screen) | N/A |
| 29 | HIV Detected - Viral Load <50 Copies/mL | <40 | 20 | Negative | AZT |
| 30 | HIV Detected - Viral Load 1,000-10,000 Copies/mL | 1760 | 2012 | Negative | Maternal triple drug ART, C-section, infant AZT prophylaxis |
| 31 | HIV Not Detected | Not Detected | Target Not Detected | Previous Known HIV Positive | N/A |
| 32 | HIV Detected - Viral Load 10,000-100,000 Copies/mL | 23600 | 26200 | Positive (Ag/Ab Combo) | Maternal triple drug ART, C-section, infant AZT prophylaxis |
| 33 | Indeterminate* | N/A | Target Not Detected | Reactive (DUO Screen) | N/A |
| 34 | HIV Detected - Viral Load 1,000-10,000 Copies/mL | 1050 | 925 | Unknown | AZT |
| 35 | HIV Detected - Viral Load <50 Copies/mL | <40 | 53 | Reactive (DUO Screen) | AZT |
| 36 | HIV Not Detected | Not Detected | Target Not Detected | Positive (Ag/Ab Combo) | N/A |
| 37 | HIV Detected - Viral Load 10,000-100,000 Copies/mL | 63100 | 77100 | Reactive (DUO Screen) | Maternal triple drug ART, C-section, infant AZT prophylaxis |
| 38 | HIV Detected - Viral Load < 50 Copies/mL | <40 | 42 | Reactive (DUO Screen) | AZT |
| 39 | HIV Detected - Viral Load < 50 Copies/mL | <40 | <20 | Reactive (DUO Screen) | AZT |
| 40 | HIV Detected - Viral Load 1,000-10,000 Copies/mL | 6710 | 5902 | Reactive (DUO Screen) | Maternal triple drug ART, C-section, infant AZT prophylaxis |
| 41 | HIV Detected - Viral Load 50-1,000 Copies/mL | 120 | 220 | Reactive (DUO Screen) | AZT |
| 42 | HIV Detected - Viral Load 50-1,000 Copies/mL | 184 | 1200 | Positive (Ag/Ab Combo) | Maternal triple drug ART, C-section, infant AZT prophylaxis |
| 43 | HIV Detected - Viral Load >100,000 Copies/mL | 122000 | 597000 | Positive (Ag/Ab Combo) | Maternal triple drug ART, C-section, infant AZT prophylaxis |
| 44 | HIV Detected - Viral Load 50 - 1,000 Copies/mL | 103 | 174 | Unknown | AZT |
| 45 | HIV Detected - Viral Load 50 - 1,000 Copies/mL | <40 | <20 | Unknown | AZT |
| 46 | HIV Not Detected | Not Detected | Target Not Detected | Not tested | N/A |
| 47 | HIV Not Detected | Not Detected | Target Not Detected | Positive (Ag/Ab Combo) | N/A |
| 48 | HIV Not Detected | Not Detected | Target Not Detected | Negative | N/A |
| 49 | HIV Not Detected | Not Detected | Target Not Detected | Negative | N/A |

*Indeterminates were referred out for further testing at RRPL and a final report. Indeterminates were not re-tested on the GeneXpert.

*Table S2*. Assay performance comparison between Cepheid GeneXpert® HIV-1 VL testing and Roche cobas® HIV-1 NAAT (VL) testing for HIV detection in female patient plasma samples. Overall, the GeneXpert® had 100% (23/23) positive agreement, 85.71% (22/26) negative agreement and 91.84% (45/49) overall agreement to Roche testing.

|  |  | **Cepheid GeneXpert® HIV-1 VL** | | |
| --- | --- | --- | --- | --- |
|  |  | Detected (n) | Not Detected (n) | Unresolved (n) |
| **Roche cobas® HIV-1 NAAT (VL)** | Positive (n) | 23 | 0 | 0 |
|  | Target Not Detected (n) | 0 | 22 | 4 |
